# Definite Differences between *In Vitro* Actin-Myosin Sliding and Muscle Contraction as Revealed Using Antibodies to Myosin Head

**DOI:** 10.1371/journal.pone.0093272

**Published:** 2014-06-11

**Authors:** Haruo Sugi, Shigeru Chaen, Takakazu Kobayashi, Takahiro Abe, Kazushige Kimura, Yasutake Saeki, Yoshiki Ohnuki, Takuya Miyakawa, Masaru Tanokura, Seiryo Sugiura

**Affiliations:** 1 Department of Physiology, School of Medicine, Teikyo University, Tokyo, Japan; 2 Department of Integrated Sciences in Physics and Biology, College of Humanities and Sciences, Nihon University, Tokyo, Japan; 3 Department of Electronic Engineering, Shibaura Institute of Technology, Tokyo, Japan; 4 Department of Physiology, School of Dentistry, Tsurumi University, Yokohama, Japan; 5 Department of Applied Biochemistry, Graduate School of Agriculture and Life Sciences, University of Tokyo, Tokyo, Japan; 6 Graduate School of Frontier Sciences, University of Tokyo, Tokyo, Japan; Semmelweis University, Hungary

## Abstract

Muscle contraction results from attachment-detachment cycles between myosin heads extending from myosin filaments and actin filaments. It is generally believed that a myosin head first attaches to actin, undergoes conformational changes to produce force and motion in muscle, and then detaches from actin. Despite extensive studies, the molecular mechanism of myosin head conformational changes still remains to be a matter for debate and speculation. The myosin head consists of catalytic (CAD), converter (CVD) and lever arm (LD) domains. To give information about the role of these domains in the myosin head performance, we have examined the effect of three site-directed antibodies to the myosin head on in vitro ATP-dependent actin-myosin sliding and Ca^2+^-activated contraction of muscle fibers. Antibody 1, attaching to junctional peptide between 50K and 20K heavy chain segments in the CAD, exhibited appreciable effects neither on in vitro actin-myosin sliding nor muscle fiber contraction. Since antibody 1 covers actin-binding sites of the CAD, one interpretation of this result is that rigor actin-myosin linkage is absent or at most a transient intermediate in physiological actin-myosin cycling. Antibody 2, attaching to reactive lysine residue in the CVD, showed a marked inhibitory effect on in vitro actin-myosin sliding without changing actin-activated myosin head (S1) ATPase activity, while it showed no appreciable effect on muscle contraction. Antibody 3, attaching to two peptides of regulatory light chains in the LD, had no significant effect on in vitro actin-myosin sliding, while it reduced force development in muscle fibers without changing MgATPase activity. The above definite differences in the effect of antibodies 2 and 3 between in vitro actin-myosin sliding and muscle contraction can be explained by difference in experimental conditions; in the former, myosin heads are randomly oriented on a glass surface, while in the latter myosin heads are regularly arranged within filament-lattice structures.

## Introduction

Although more than 50 years have passed since the monumental discovery that muscle contraction results from relative sliding between actin and myosin filaments coupled with ATP hydrolysis [1], [2], molecular mechanisms of the myofilament sliding are not yet fully understood. It is generally believed that a myosin head extending from myosin filaments first attaches to actin filaments, undergoes conformational changes to produce unitary myofilament sliding, and then detaches from actin filaments [3], [4]. In accordance with this view, biochemical studies on reaction steps of actomyosin ATPase in solution [5] indicate that the myofilament sliding is caused by cyclic interaction between myosin heads and actin filaments; the myosin head (M) first attaches to actin (A) in the form of M⋅ADP⋅Pi to undergo a conformational change, i.e. power stroke, associated with release of Pi and ADP, and then forms rigor linkage (AM) with A. Upon binding with a new ATP, M detaches from A to exert a recovery stroke associated with formation of M⋅ADP⋅Pi to again attach to A. Despite extensive studies, the myosin head power stroke still remains to be a matter for debate and speculation [6].

The myosin head (myosin subfragment 1, S1) consists of catalytic domain (CAD) and lever arm domain (LD), which are connected by converter domain (CVD). In 1989, Sutoh, Tokunaga and Wakabayashi [7] prepared monoclonal antibodies; one directed to junctional peptide between 50-kDa and 20 kDa heavy chain segments in the CAD (anti-CAD antibody), while the other directed to reactive lysine residue (Lys83) located close to the junction between the CAD and the CVD (anti-RLR antibody) [8], and succeeded in showing that anti-CAD antibody binds at the distal region of the CAD, while anti-RLR antibody binds at the boundary between the CAD and CVD domains. The gas environmental chamber (EC) enables us to study dynamic structural changes of hydrated biomolecules electron microscopically. Using the EC, we succeeded in recording ATP-induced movement of individual myosin heads, effectively position-marked with anti-CAD or anti-RLR antibody, in hydrated vertebrate myosin filaments in the absence of actin filaments [9], [10], [11]. On ATP application, myosin heads moved away from the central bare region of myosin filaments with an amplitude of 5–7.5 nm, and after exhaustion of applied ATP, myosin heads returned towards their initial position, indicating our success in visualizing myosin head recovery stroke [10]. More recently, we have further succeeded in recording myosin head power stroke in hydrated mixture of actin and myosin filaments [12], [13]. These results constitute the first electron microscopic visualization of myosin head power and recovery strokes producing muscle contraction.

In the present study, we attempted to examine whether or not these site-directed antibodies have influence on ATP-dependent actin-myosin sliding and Ca^2+^-activated muscle contraction. In addition to anti-CAD and anti-RLR antibodies, we also used another antibody directed to junctional peptides of two regulatory light chains in the LD [11]. Unexpectedly, we have obtained the following results : (1) anti-CAD antibody showed effects neither on in vitro actin-myosin sliding nor on muscle fiber contraction; (2) anti-RLR antibody inhibited in vitro actin-myosin sliding without affecting actin-activated S1 ATPase activity, but had no effect on muscle fiber contraction; and (3) anti-LD antibody showed no significant effect on in vitro actin-myosin sliding, but reduced Ca^2+^-activated isometric force development without changing the maximum velocity of shortening and MgATPase activity. One interpretation of these results is that, during muscle contraction, rigor actin-myosin linkage AM is absent or at most a transient intermediate. It is also suggested that the mechanism of in vitro actin-myosin sliding definitely differs from muscle contraction, due to random orientation and fixation of myosin heads in vitro motility assay systems [14].

## Materials and Methods

### Preparation of Monoclonal Antibodies to Myosin Head

**Figure 1:**
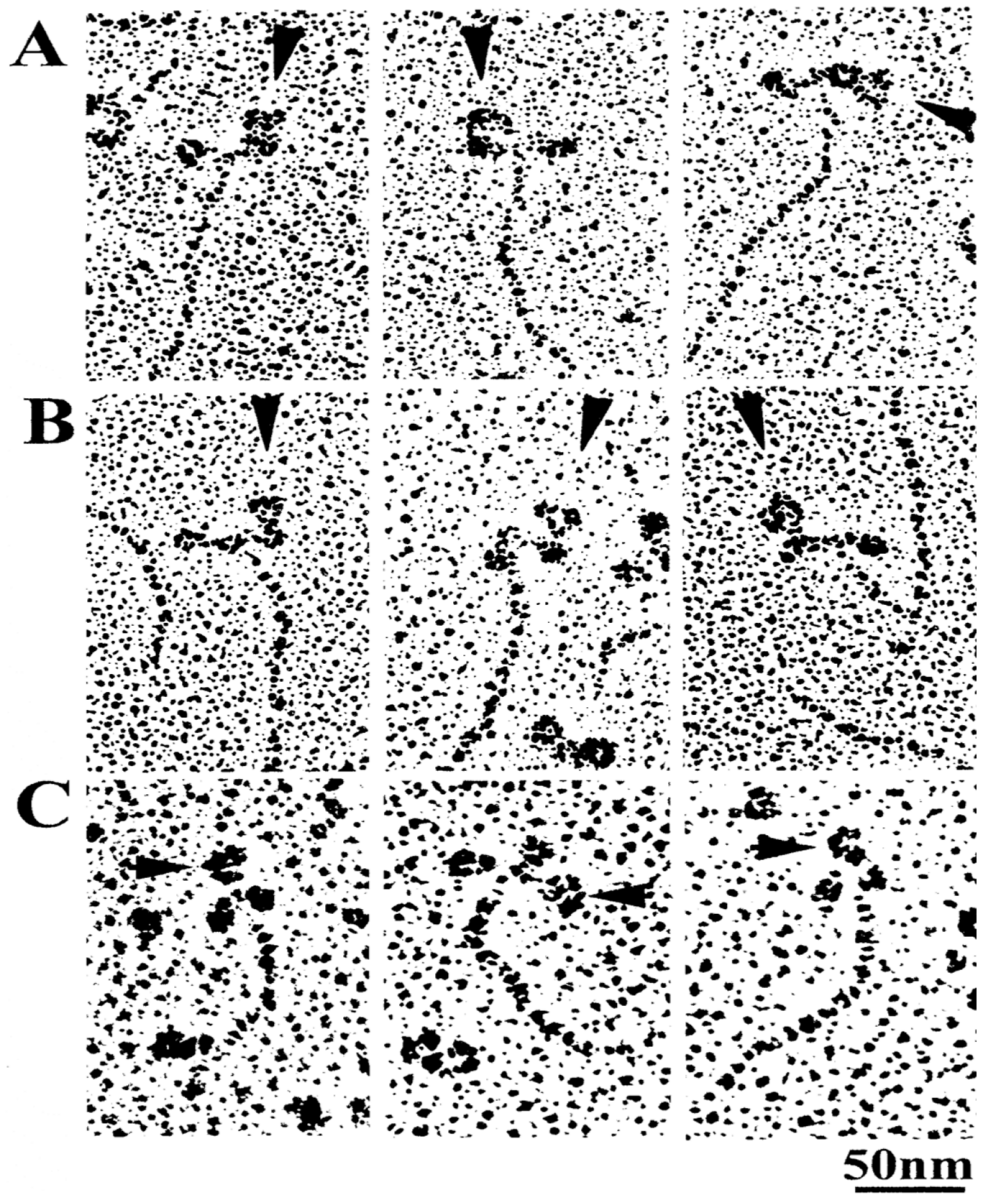
A gallery of electron micrographs of rotary shadowed antibodies (IgG) attached to myosin heads. Anti-CAD antibody-myosin(A), anti-RLR antibody-myosin (B), and anti-LD antibody-myosin (C) complexes are shown in A,B and C, respectively. IgG molecules are indicated by arrowheads. The panels are provided from the published papers (Sutoh et al., 1989; Minoda et al., 2011).

Monoclonal antibodies (IgG) directed to the junctional peptide between 50- and 20-kDa segments of myosin heavy chain, located close to the actin binding sites (anti-CAD antibody), and directed to the reactive lysine residue (lys83), located close to the CAD-CVD junction (anti-RLR antibody) were prepared by the method of Sutoh et al. [7]. Monoclonal antibody (IgG) directed to two peptides (Met58∼Ala70 and Leu106∼Phe120) of the myosin regulatory light chain in the LD (anti-LD antibody) was prepared by the method of Minoda et al. [11]. As shown in Fig. 1, anti-CAD antibody and anti-RLR antibody molecules (IgG) actually attach to the head of myosin molecule (A and B), while anti-LD antibody molecules (IgG) attach to the head-rod junction of myosin molecule, i.e. the myosin LD (C). These antibodies have already been proved to effectively position-mark myosin heads in synthetic myosin filaments [9], [10], [11].

### Measurement of *In Vitro* Sliding Velocity of Actin Filaments on Myosin Heads

Myosin and actin were prepared from rabbit skeletal muscle by the method of Perry [15] and Spudich and Watt [16], respectively. First, myosin in a high salt solution, containing 0.6 M KCl, 50 mM phosphate buffer (pH 6.8) and 0.1 mM DTT, was introduced at a concentration of 0.1 mg/ml into a flow chamber. The chamber was then rinsed with 1 mg/ml BSA, and assay solution containing 10 nM F-actin filaments, labeled with rhodamine-conjugated phalloidin (Molecular Probe), 25 mM KCl, 25 mM imidazole (pH 7.5), 2 mM ATP, 4 mM MgCl_2_, 1 mM DTT, 1% β-mercaptoethanol, 4.5 mg/ml glucose, 0.21 mg/ml glucose oxidase, 0.035 mg/ml catalase, and anti-CD antibody or anti-LD antibody was introduced into a nitrocellulose-coated flow cell. Sliding velocity of actin filaments on myosin heads was determined using a video system (30 frames/s, Hamamatsu Photonics C-7190). Experiments were made at 25°C.

### Measurement of Actin-Activated S1 MgATPase Activity

Myosin subfragment 1 (S1) was obtained by digestion of myosin with chymotrypsin following the procedure of Margossian and Lowey [17]. Actin-activated myosin head (S1) Mg-ATPase activity was determined by mixing S1 (0.05 mg/ml) with F-actin (0–50 µM) and measuring release of phosphate (Pi) at 25°C using the malachite green method [18], in an assay buffer containing 25 mM KCl, 25 mM imidazole (pH 7.5), 4 mM MgCl_2_, 1 mM DTT. Reaction was initiated by adding 1/5 volume of 6 mM ATP, and stopped by adding 4 volumes of 0.3 M perchloric acid. Data from 2–3 independent S1 samples were averaged.

### Determination of Contraction Characteristics of Ca^2+^-Activated Muscle Fibers

White male rabbits weighing 2 to 2.5 kg were killed by injection of sodium pentobarbital (50 mg/kg) into the ear vein, and psoas muscles were dissected from the animals. The animals were treated in accordance with the Guiding Principles for the Care and Use of Animals in the Field of Physiological Sciences, published by the Physiological Society of Japan. The protocol was approved by the Teikyo University Animal Care Committee (protocol #07-050). Glycerol-extracted muscle fiber strips were prepared from rabbit skeletal muscle as described by Sugi et al. [19]. Single muscle fibers (diameter, 50–80 µm) were dissected from the glycerol-extracted strips, and mounted horizontally in an experimental chamber (0.1 ml) between a force transducer and a servomotor by glueing both ends to the extension of the transducer and the servomotor with collodion. The servomotor contained a displacement transducer (differential capacitor) sensing the motor arm position. Further details of experimental apparatus have been described elsewhere [18]. The fibers were kept at their slack length (L_0_, sarcomere length 2.4 µm). Relaxing solution contained 125 mM KCl, 4 mM MgCl_2_, 4 mM EGTA, 20 mM Pipes (pH 7.0). Contracting solution was prepared by adding 4 mM CaCl_2_ to relaxing solution to maximally activate the fibers. The experiments were made at 20°C.

The servomotor system was operated either in the length clamp mode or in the force control mode [20]. First, the system was in the length clamp mode so that the fiber contracted isometrically in contracting solution. After the fiber developed steady isometric force, the servomotor system was switched to the force control mode, and a ramp decrease in force ( = load) from the steady force to zero (complete in 50–100 ms) was applied to the fiber. The resulting fiber shortening was recorded together with the ramp decrease in force, and the force-velocity curve was obtained and displayed on the X-Y plotter. Details of the method have been described elsewhere [19], [20], [21].

The fiber was first activated maximally with contracting solution, and when the maximum steady isometric force was developed, the force-velocity curve was obtained by applying a ramp decrease in force. Then the fiber was made to relax in relaxing solution containing the antibody, and after 30 min it was again activated with contracting solution containing the antibody.

### Measurement of MgATPase Activity of Ca^2+^-activated Muscle Fibers

Mg-ATPase activity of a small fiber bundle consisting of 2–3 muscle fibers during Ca^2+^ -activated isometric force development was recorded by the decrease of NADH during cleavage of ATP [19], [22]. The fibers were mounted in the sample compartment (0.36 ml) of a dual wavelength spectrophotometer (Nihon Bunko) with a sample monochrometer at 340 nm and a reference monochrometer at 400 nm, so that the decrease of NADH was measured from the difference in absorbance between 340 and 400 nm. To both relaxing and contracting solutions, 0.25 mM NADH, 1.25 mM phosphoenolpyruvate, 50 units/ml pyruvate kinase, 50 units/ml lactic dehydrogenase, 10 mM NaN_3_, 50 µM quercetin, 1 µg/ml oligomycin were added. Solutions in the compartment was constantly stirred with a magnetic stirrer. The experiments were performed at 10°C.

## Results

### Anti-CAD Antibody Has No Effect on Both *In Vitro* Actin-Myosin Sliding and Muscle Fiber Contraction

**Figure 2:**
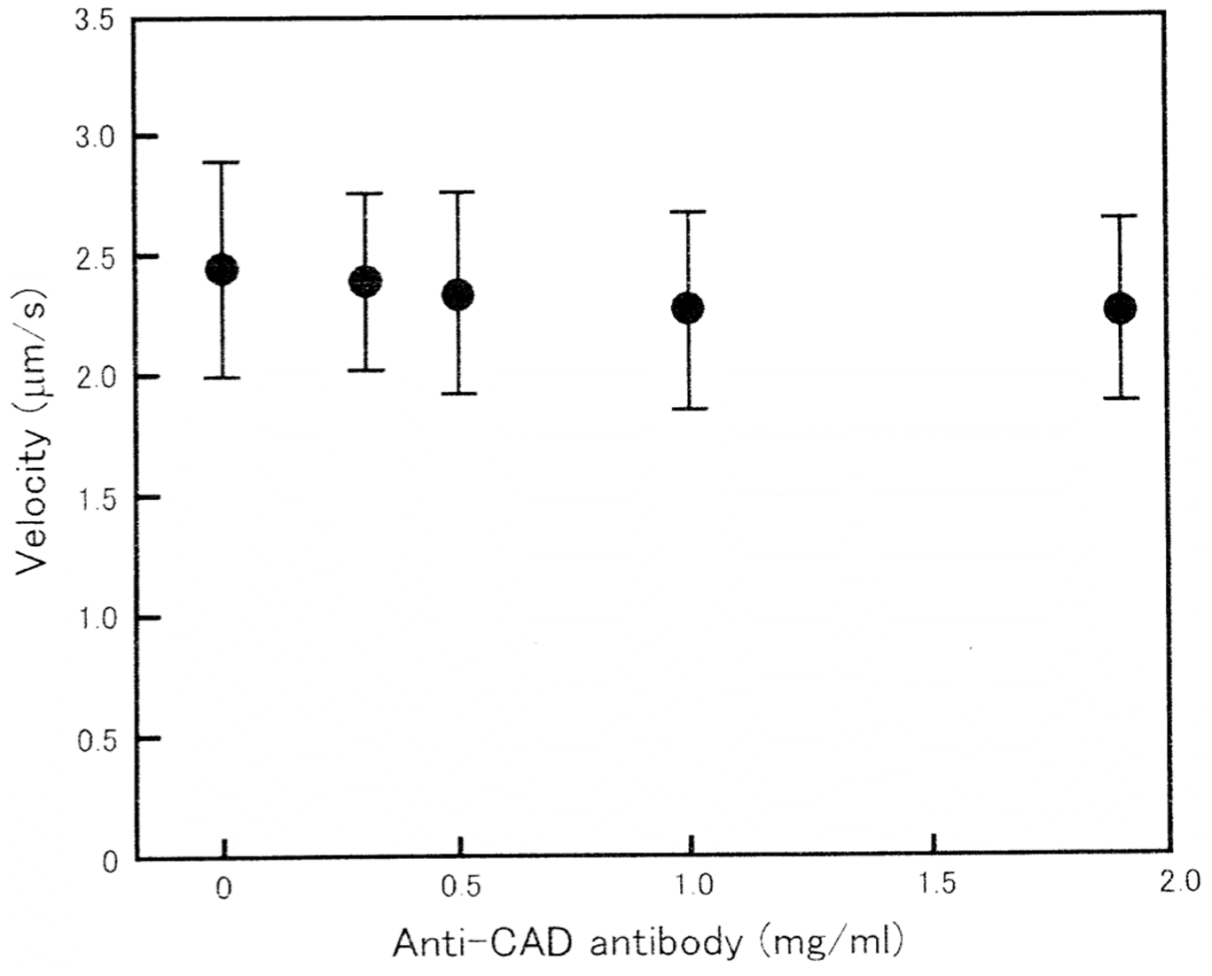
No appreciable effect of anti-CAD antibody on *in vitro* actin filament sliding on myosin heads. In this and Figs. 4 and 5, mean velocities of sliding are plotted against antibody concentration with vertical bars indicating S.D., and each data point is obtained from 80–120 measurements.

Prior to the application of anti-CAD antibody, the average velocity of ATP-dependent in vitro actin filament sliding on myosin heads was determined to be 2.3±0.6 µm/s (mean±SD, n = 380 from 10 independent experiments). Though this value is much smaller than that reported by Harada et al. [23], it has been pointed out by Uyeda et al. [24] that the velocity of actin-myosin sliding is markedly influenced by a number of factors, such as preparation of myosin sample, composition of substratum on which actin-myosin sliding takes place, and density of myosin heads in the assay system. As shown in Fig. 2, the velocity of in vitro actin-myosin sliding did not change appreciably in the presence of anti-CAD antibody at concentrations up to 2 mg/ml. Similar results were obtained from 9 other experiments.

**Figure 3:**
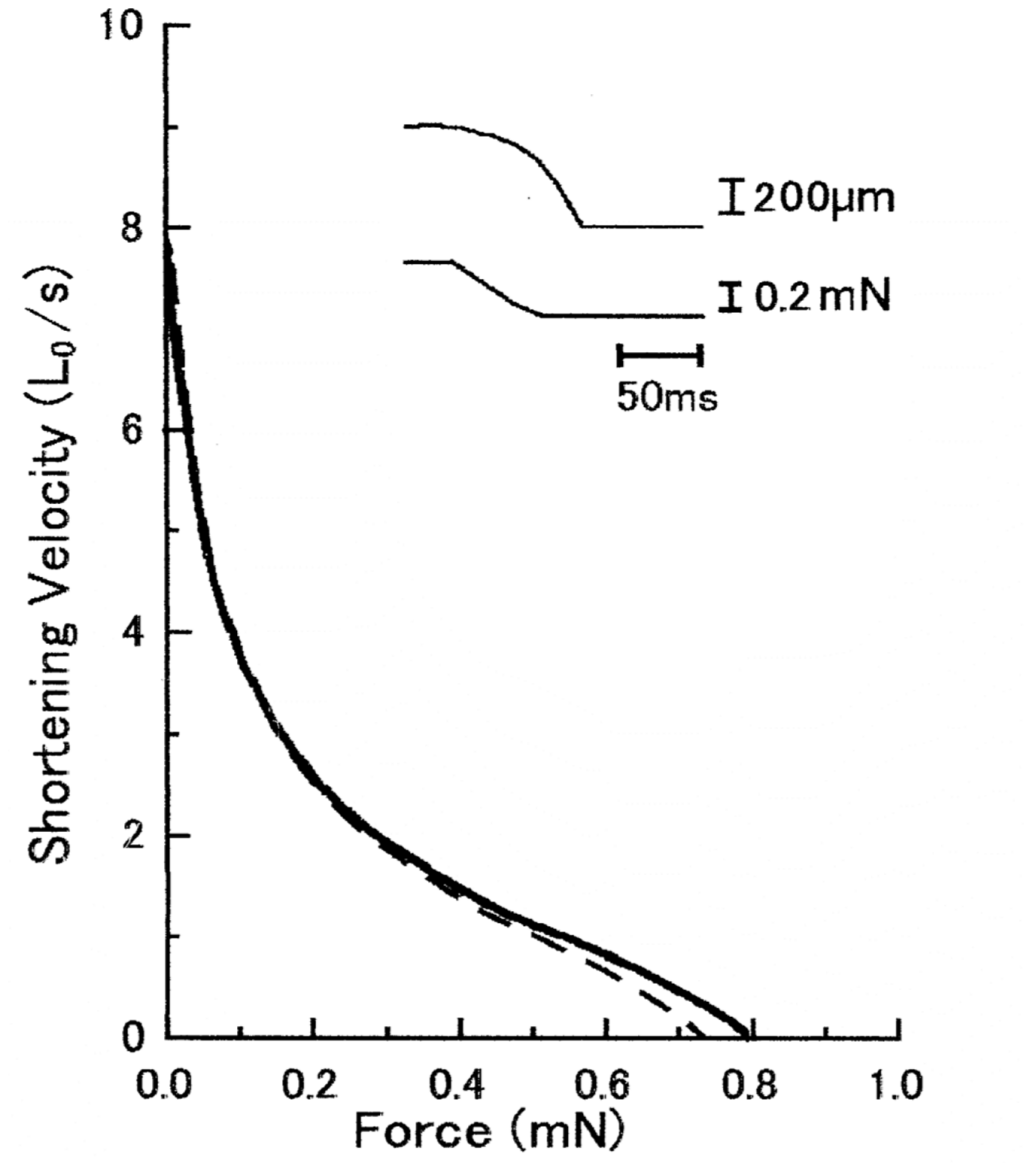
No appreciable effect of anti-CAD antibody on force-velocity curves of Ca^2+^-activated muscle fiber. In this and Fig. 5, solid and broken lines indicate force-velocity curves before and after application of anti-CAD antibody (2 mg/ml), respectively. Inset shows an example of fiber length (upper trace) and force (lower trace) changes in response to a ramp decrease in force.

Fig. 3 shows superimposed force-velocity curves of a maximally Ca^2+^-activated muscle fiber, obtained in the absence and in the presence of anti-CAD antibody at concentrations up to 2 mg/ml. As can be seen in the figure, the two curves were double-hyperbolic in shape [19], [21], [25], and almost identical with each other. The maximum shortening velocity at zero external load, i.e. the point of intersection of the force-velocity curve with the velocity axis, remained unchanged in the presence of antibody; the small difference in the maximum isometric force was well within the range of variation in skinned fiber preparations [26].

### Anti-RLR Antibody Inhibits *In Vitro* Actin-Myosin Sliding, but Has No Effect on Muscle Fiber Contraction

**Figure 4:**
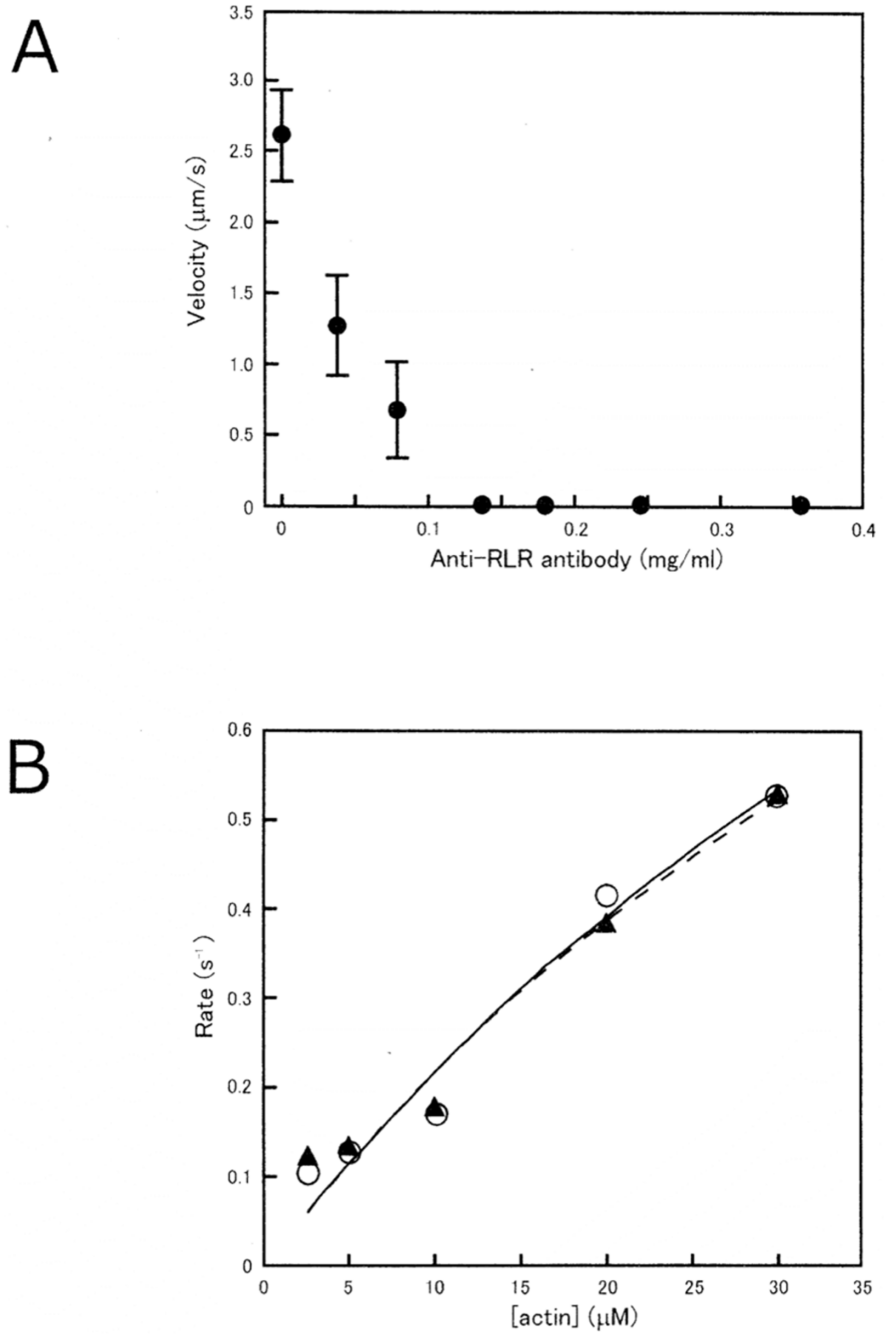
(A) Inhibitory effect of anti-RLR antibody on in vitro actin filament sliding on myosin heads. (B) No appreciable effect of anti-RLR antibody on actin-activated myosin head (S1) MgATPase activity. In B, the ATPase activities in the absence and in the presence of the antibody (0.36 mg/ml) are shown by open circles and black triangles, respectively. Curves are fitted by Michaelis-Menten kinetics.

As shown in Fig. 4A, anti-RLR antibody showed a marked inhibitory effect on ATP-dependent in vitro actin-myosin sliding. The velocity of actin filament sliding on myosin heads decreased by about 50% with 0.02 mg/ml antibody, and the actin-myosin sliding was completely eliminated with the antibody >1.4 mg/ml. Similar results were obtained on 7 other experiments. On the other hand, actin-activated MgATPase activity of myosin head (S1) did not change appreciably in the presence of anti-RLR antibody >1.4 mg/ml; both the V_max_ (1.98 s^−1^) and the K_m_ (81.6 µM) remained unchanged by the antibody (Fig. 4B).

**Figure 5:**
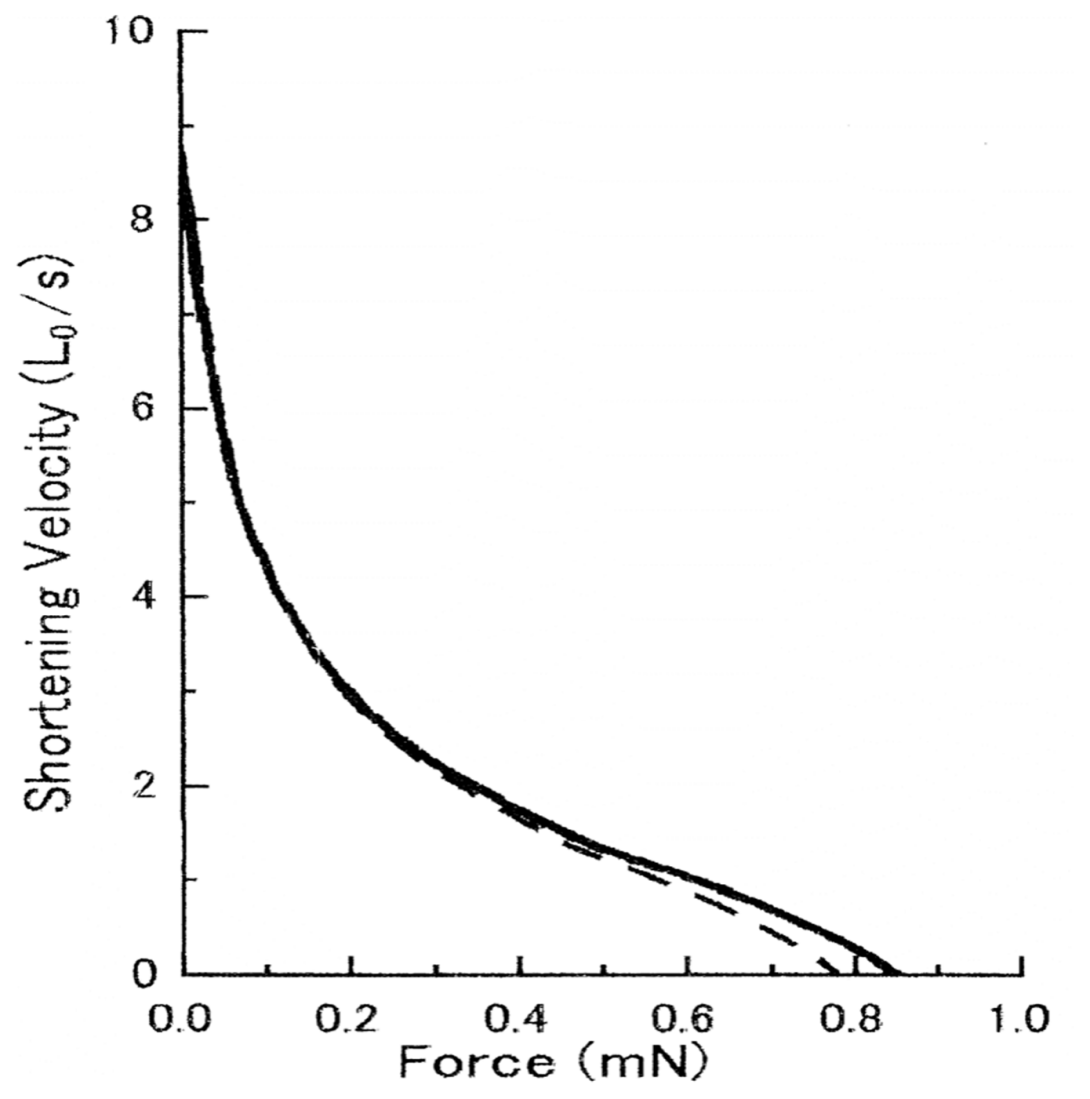
No appreciable effect of anti-RLR antibody on force-velocity curves of Ca^2+^-activated muscle fiber.

In contrast with its marked inhibitory effect on in vitro ATP-dependent actin-myosin sliding, anti-RLR antibody showed no appreciable effect on contraction of Ca^2+^-activated muscle fibers at concentrations up to 2 mg/ml. As has been the case with anti-CAD antibody, the force-velocity curves in the presence and absence of anti-RLR antibody were almost identical, so that both the maximum velocity of shortening V_max_ and the maximum isometric force remained unchanged in the presence of anti-RLR antibody (Fig. 5). Similar results were obtained on 8 other experiments obtained from 8 different fibers.

### Anti-LD Antibody Has No Significant Effect on *In Vitro* Actin-Myosin Sliding, but Inhibits Ca^2+^-Activated Force Development of Muscle Fibers without Affecting MgATPase Activity

**Figure 6:**
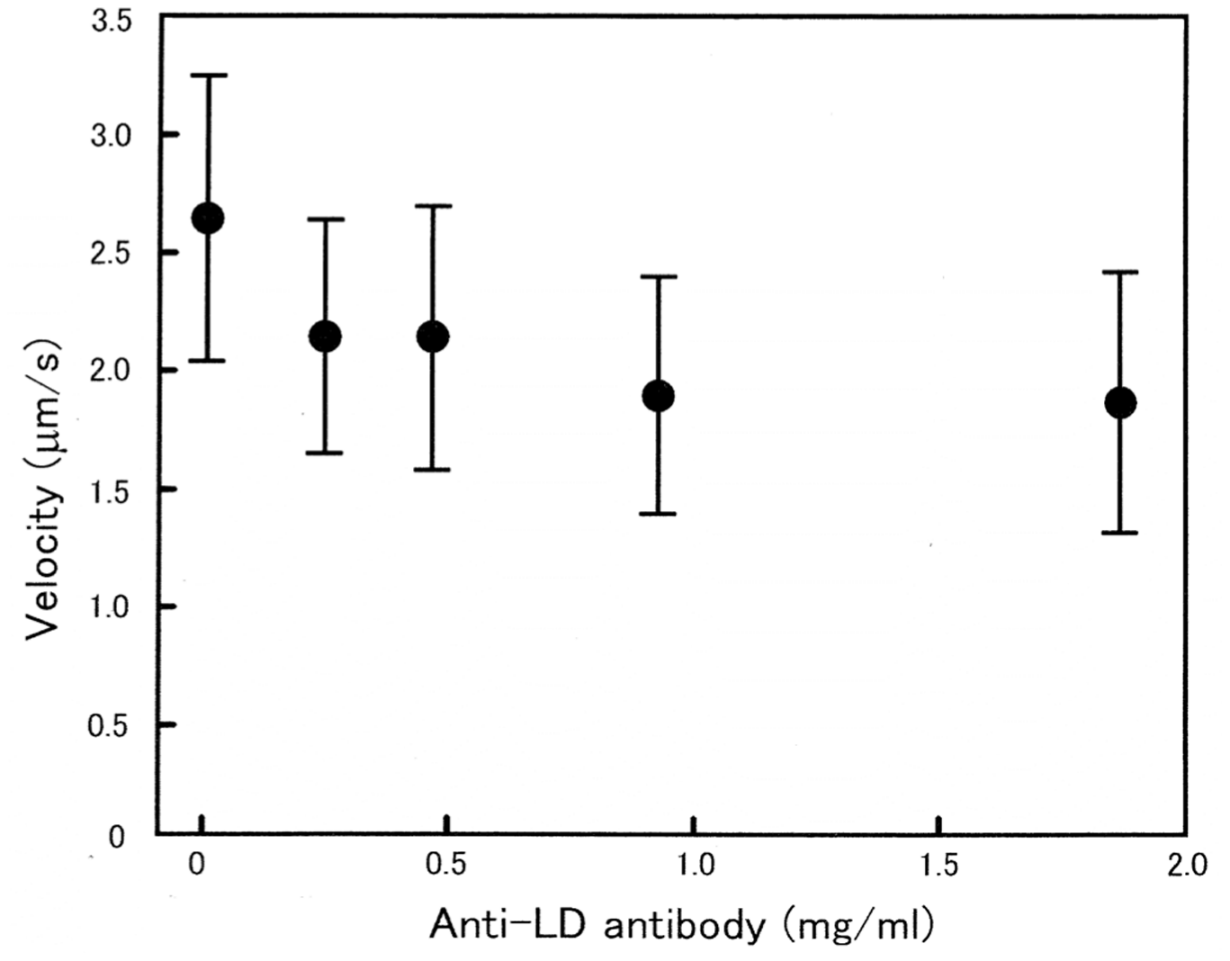
No appreciable effect of anti-LD antibody on *in vitro* actin filament sliding on myosin heads.

As shown in Fig. 6, the sliding velocity of actin filaments on myosin heads did not change significantly in the presence of the antibody up to 2 mg/ml. Though the velocity of actin filament sliding exhibited a tendency to decrease slightly with increasing antibody concentration, the difference between the data points was not statistically significant. Similar results were obtained from 6 other experiments.

**Figure 7:**
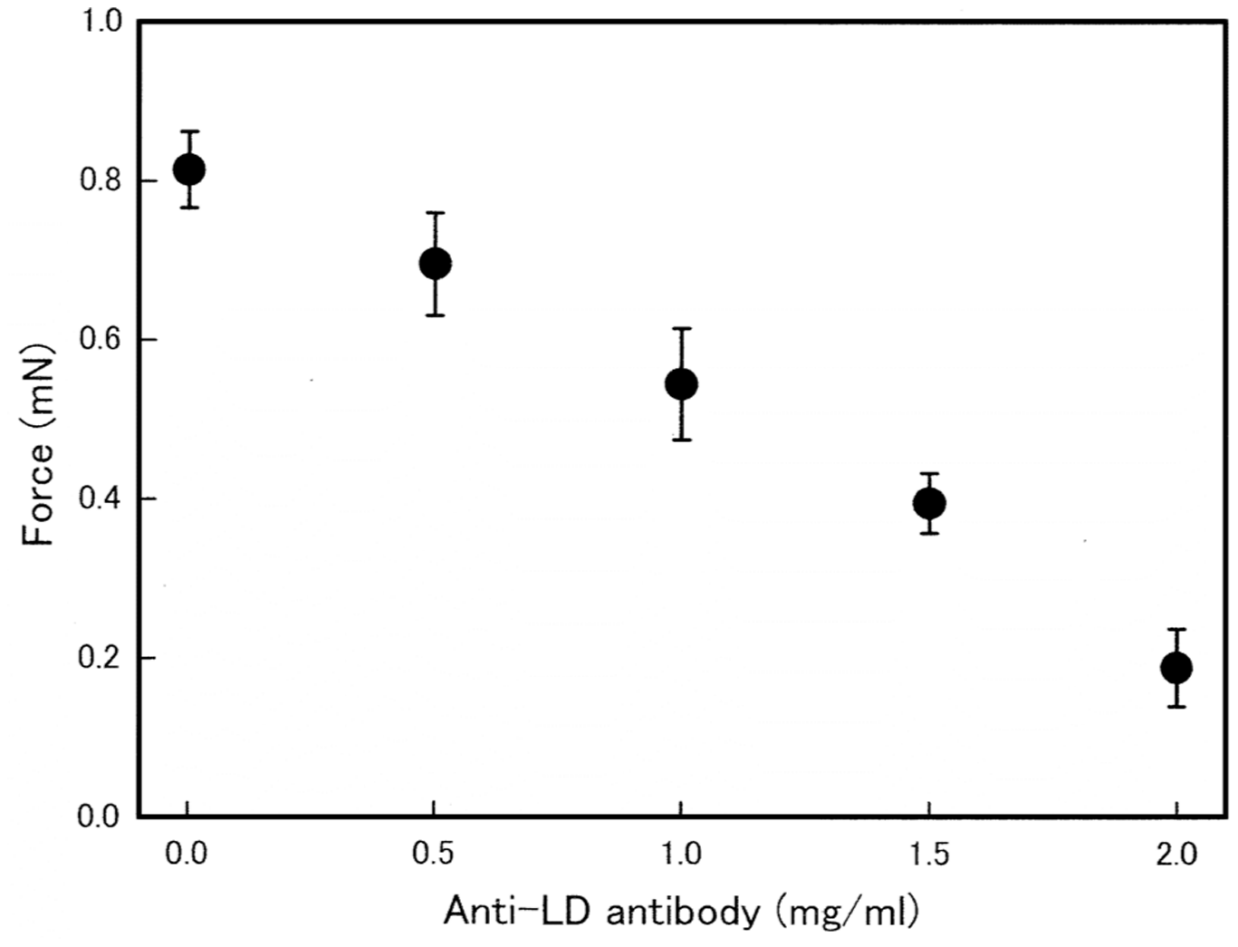
Inhibitory effect of anti-LD antibody on Ca^2+^-activated isometric force development. The magnitude of steady Ca^2+^-activated isometric force is plotted against the antibody concentration. Vertical bars represent S.E.M. (n = 4∼6).

**Figure 8:**
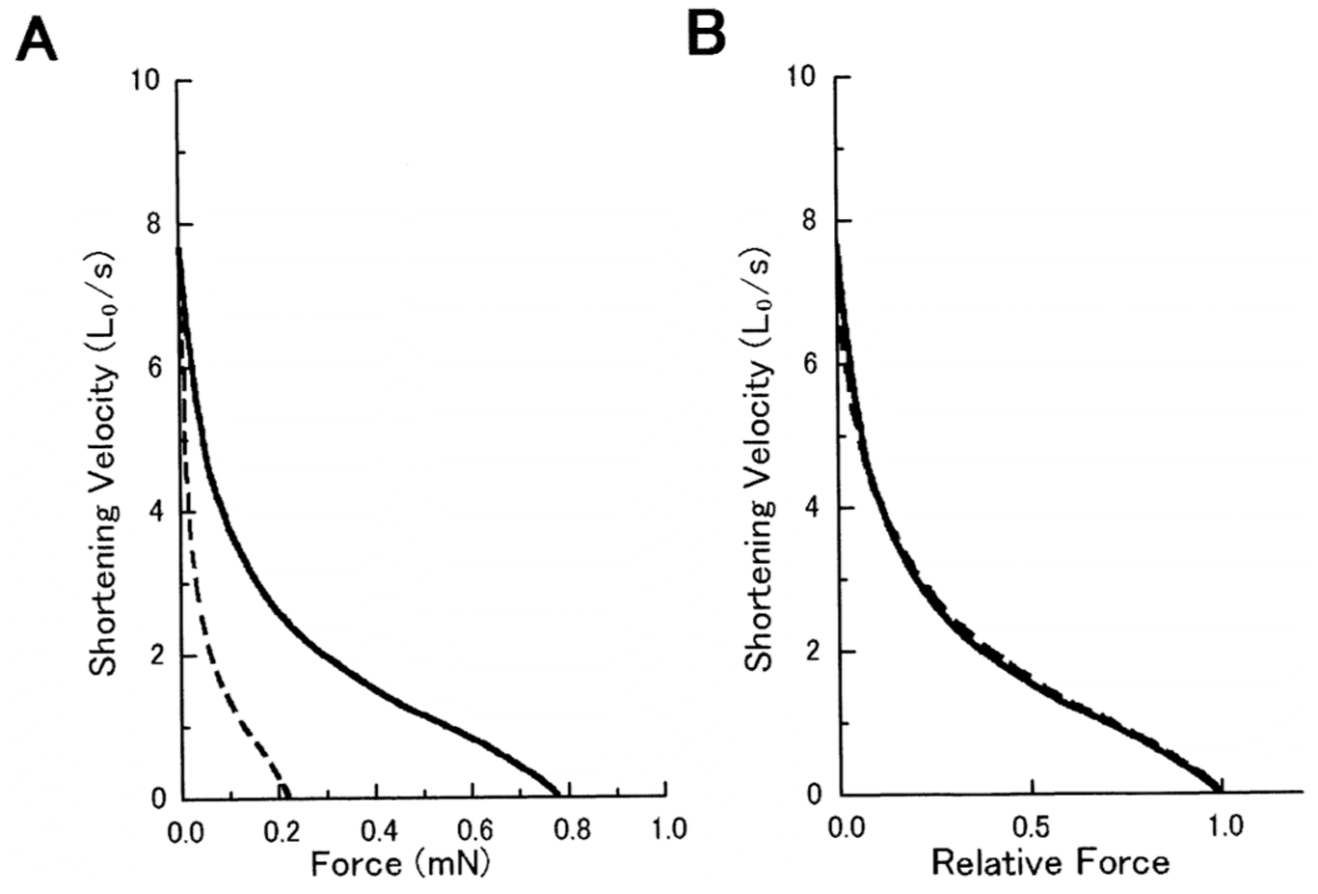
Effect of anti-LD antibody on force-velocity curves of a Ca^2+^-activated muscle fiber. (A) Force-velocity curves before (solid line) and after (broken line) application of the antibody (1.5 mg/ml). (B) The same force-velocity curves with force expressed relative to the maximum value.

In contrast, anti-LD antibody was found to inhibit Ca^2+^-activated isometric force development of muscle fibers in a concentration-dependent manner (Fig. 7). In the presence of 2 mg/ml anti-LD antibody, Ca^2+^-activated isometric force was reduced by 70–80%. The effect of anti-LD antibody was reversible. When the fibers were returned to relaxing solution without antibody and kept in it for 20 min, they completely restored their ability to develop Ca^2+^-activated isometric force as large as that before application of the antibody. Examples of the force-velocity curves obtained in the absence and in the presence of the antibody obtained from one and the same fiber are presented in Fig. 8. Despite a marked decrease in Ca^2+^-activated isometric force in the presence of the antibody (2 mg/ml), the V_max_ remained unchanged (Fig. 8A), and if the force was normalized with respect to the maximum values, the two curves were found to be identical (Fig. 8B).

**Figure 9:**
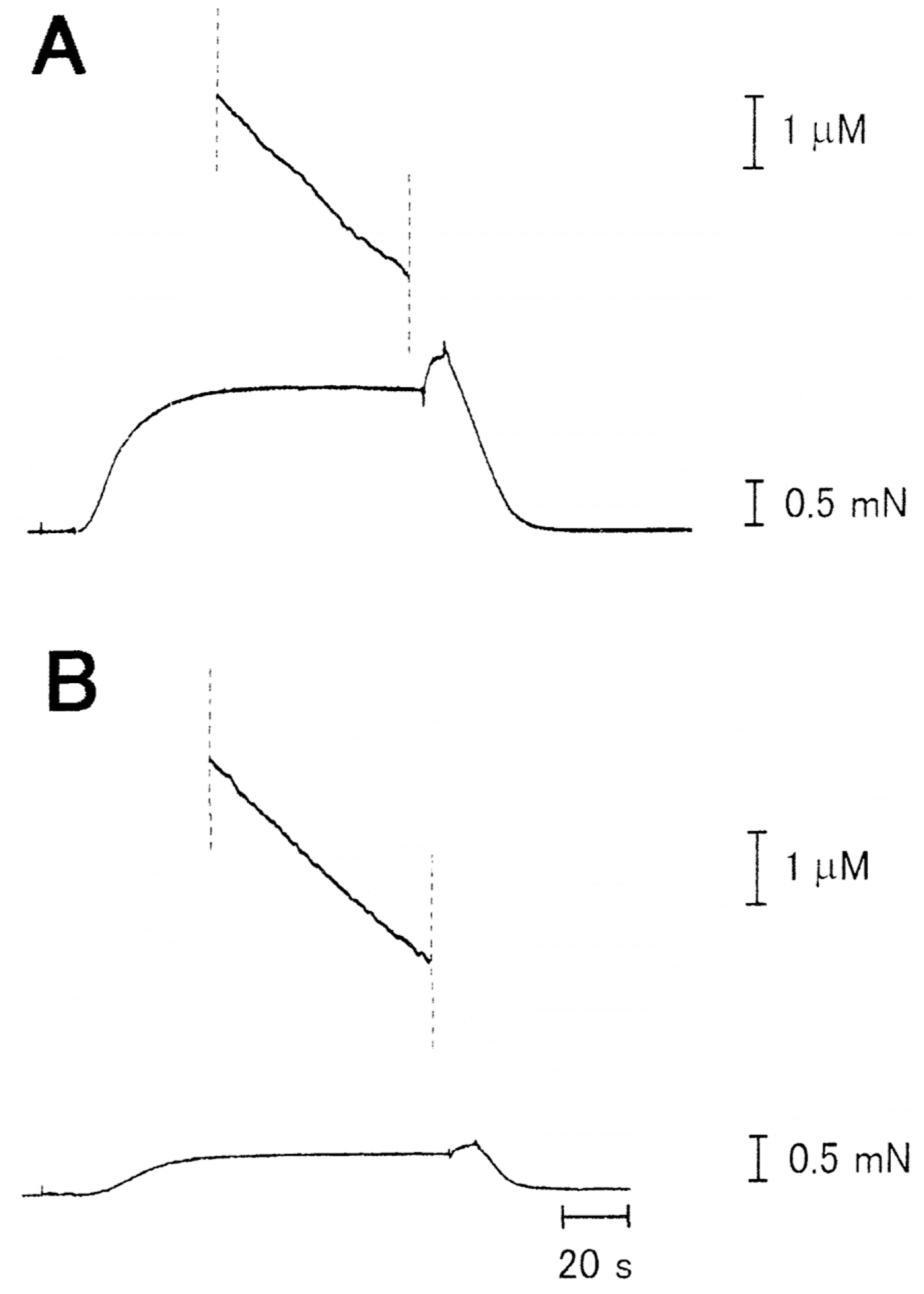
Simultaneous recordings of MgATPase activity (upper traces) and Ca^2+^-activated isometric force development (lower traces) of a small bundle consisting of two fibers. Records A and B are taken before and after application of anti-LD antibody (2 mg/ml), respectively.

Fig. 9 shows typical examples of simultaneous recordings of MgATPase activity and isometric force development of a Ca^2+^-activated muscle fiber. Despite the marked reduction of isometric force, MgATPase activity of the fiber, as measured from the slope of ATPase records during the steady isometric force development, did not change significantly. In 8 different fibers examined, the MgATPase activity in the absence and in the presence of the antibody (2 mg/ml) was 0.55±0.17 mM/s and 0.54±0.16 mM/s (mean±SD, n = 8), respectively.

## Discussion

### Evaluation of the Proportion of Myosin Heads Bound to Antibodies in the Present Experiments

Prior to the discussion on the effect of antibodies on in vitro actin-myosin sliding and muscle fiber contraction, it seems necessary to evaluate the proportion of myosin heads bound to antibodies in the present experiments, especially within single skinned muscle fibers. First of all, the possibility that antibodies do not diffuse into muscle fibers can be excluded by the following reasons. (1) Using the confocal microscope, fluorescently labeled dummy antibody (IgG) has been shown to diffuse into skinned muscle fibers, and evenly distribute in the interior of the fiber within 3 min [27]. (2) Anti-LD antibody, which is believed to differ from anti-CAD antibody only at the epitope-binding site, shows reversible inhibitory effect on Ca^2+^-activated force development (Figs. 4–7), indicating that IgG molecules can diffuse into the fiber to exhibit their inhibitory effect by attaching to their epitope, and can diffuse out of the fiber after its removal from external solution. The same explanation may apply to both anti-CAD antibody and anti-RLR antibody, which has no effect on Ca^2+^-activated contraction of muscle fibers. (3) In addition, we have recently found that development of rigor force and stiffness in skinned fibers is reversibly slowed down, without appreciably changing their peak values, by all the three antibodies used in the present study [28], again indicating that these antibodies can diffuse into the fiber to bind with their respective epitope. In the case of anti-CAD antibody, its ineffectiveness in inhibiting rigor linkage formation might result from that the antibody (IgG) molecules might first attach to myosin heads, but then would gradually be overridden by actin filaments which tend to form strong and stable rigor linkages with myosin heads.

On the other hand, our published electron micrographs of hydrated synthetic myosin filaments [10, Fig. 2] give information about the proportion of myosin heads bound with antibodies within a single muscle fiber. Judging from the structure of myosin filaments consisting of myosin molecules, the number of myosin heads in each native myosin filament (diameter, 15 nm, length, 1.6 µm) is estimated to be 300. The synthetic myosin filament (myosin-myosin rod copolymer mixed at a ratio of 1∶1) shown in Fig. 2B [10] has a length of ∼1.5 µm and a diameter of ∼90 nm. As a native myosin filament is 1.6 µm in length and 15 nm in diameter, the size of this particular filament roughly corresponds to a bundle consisting of 6 native myosin filaments running in parallel with one another. Considering that this filament is a 1∶1 mixture of myosin and myosin rod, the number of myosin heads in it may be ∼900 (300×6×1/2). Since antibodies can attach only one of the two myosin heads in each myosin molecule [10], the number of myosin heads available for antibodies is ∼450. In addition, it seems likely that myosin heads located at the filament surface can only bind with externally applied antibodies. If this factor is taken into consideration, the number of myosin heads available for antibodies would be further reduced to <200. In fact, close inspection of the synthetic filament shown in Fig. 2B [10] indicates that the number of gold particles attached to myosin heads via anti-CAD antibody is >200. This may be taken to indicate the high affinity of anti-CAD antibody to myosin head. In the case of a large synthetic filament shown in Fig. 2A [10], however, the number of gold particles attached to myosin heads is not so large, probably because antibodies may not readily accessible to myosin heads due to complex myosin-myosin rod network. We made similar observations on electron micrographs of synthetic filaments, in which myosin heads were position-marked with anti-RLR and anti-LD antibodies [11].

In conclusion, in the case of muscle fibers containing regularly arranged myofilament-lattice, the proportion of myosin heads with bound antibody may be expected to be fairly large, probably at least >50%. The same conclusion may also apply to myosin heads fixed on nitrocellulose membrane.

### Evidence for the Absence of Rigor Linkages during Cyclic Actin-myosin Interaction in Muscle Contraction

A most striking result found in the present study is that anti-CAD antibody has appreciable effects neither on ATP-dependent in vitro actin-myosin sliding nor Ca^2+^-activated muscle fiber contraction (Figs. 2 and 4). It has been known that myosin heads have two actin-binding sites, one at residues 700∼720 in the 20-kDa segment, while the other at residues towards the C-terminus of the 50-kDa segment of myosin heavy chain. Both binding sites are close to the junctional peptide connecting these two heavy chain segments [29]. If bulky anti-CAD antibody (IgG) binds to the junctional peptide, the two actin-binding sites are completely covered by the antibody molecule, so that myosin heads can no longer form linkages with the myosin-binding sites on actin monomers constituting actin filaments. As already mentioned in this paper, it seems possible that, during cyclic actin-myosin interaction taking place in muscle, rigor myosin head AM may be absent or at most a transient intermediate. This idea is inconsistent with the general view [5] that myosin heads take rigor or rigor-like configuration at the end of their power stroke during muscle contraction.

In this connection, Radocaj et al. [30] attempted to detect rigor or rigor-like myosin heads with the technique of time-resolved X-ray diffraction. They applied ramp-shaped releases to Ca^2+^-activated skinned muscle fibers, in the hope that myosin heads at the end of their power stroke would accumulate transiently. They failed, however, to detect accumulation of rigor myosin heads AM. They suggest that, at the end of power stroke, myosin heads would take the form of AM, which is structurally different from the rigor actin-myosin complex, determined by static crystallographic and electron microscopic studies [6]. In accordance with their suggestion, we have recently obtained evidence that myosin heads in rigor muscle fibers contain two different types of rigor AM linkages, one with somewhat active mechanical property while the other with purely passive property [31]. The former active AM linkages have a long average lifetime, and it seems possible that myosin heads mostly take the form of this active AM linkages during physiological actin-myosin interaction. Of course, much more experimental work is needed to settle this important issue.

### Definite Differences Between *In Vitro* Actin-Myosin Sliding and Muscle Fiber Contraction

Homsher et al. [32] compared the effect of substrate concentration, ionic strength and temperature on the velocity of in vitro ATP-dependent actin-myosin sliding (V_f_) and on the maximum unloaded shortening velocity of Ca^2+^-activated muscle fibers (V_u_). They found that these factors affected both V_f_ and V_u_ in a qualitatively similar manner except for extreme conditions. In contrast, the present study has revealed striking differences in the effect of anti-RLR and anti-LD antibodies between in vitro actin-myosin sliding and muscle fiber contraction. Anti-RLR antibody showed a marked inhibitory effect on in vitro actin-myosin sliding without changing actin-activated myosin head (S1) MgATPase activity (Fig. 4A,B), but had no appreciable effect on Ca^2+^-activated muscle fiber contraction (Fig. 5). The marked inhibitory effect of anti-RLR antibody on in vitro actin-myosin sliding (Fig. 4A) is consistent with the report of Muhlrad et al. [33] that chemical modification (trinitrophenylation) of the RLR inhibits in vitro actin-myosin sliding. They showed, however, that the chemical modification of the RLR eliminated actin-activated myosin head (S1) MgATPase activity, while in the present study it was not affected by the antibody (Fig. 4B). It may be that the chemical modification of the RLR may change 3D structure of myosin heads to inhibit the ATPase activity of the CAD, whereas the reversible binding of anti-RLR antibody to the RLR do not produce any structural changes in the CAD.

The striking difference in the effect of anti-RLR antibody between in vitro actin-myosin sliding and muscle fiber contraction can be accounted for in terms of difference in the condition, under which myosin heads interact with actin filaments. In the in vitro systems, myosin heads are randomly oriented on a glass surface, and each myosin head should have enough flexibility to interact with an actin filament moving in random directions; if the RLR binds to anti-RLR antibody, or is chemically modified [33], the flexibility of myosin heads around the CVD would be markedly reduced to impair their ability to interact with actin filaments. Meanwhile, in muscle fibers, each myosin filament is surrounded by 6 actin filaments at appropriate distances in the hexagonal myofilament-lattice structure, so that myosin heads on myosin filaments can interact with actin filaments despite the reduced flexibility around the CVD caused by binding of anti-RLR antibody. The explanation stated above is supported by our unpublished observation that the sliding velocity of fluorescently labeled actin filaments along synthetic “mini” myosin filaments (length, ∼500 nm) [34] is 2.36±1.26 µm/s (mean±SD, n = 211, at 25°C), and does not change appreciably in the presence of anti-RLR antibody (up to 2 mg/ml), since myosin heads in the filament are regularly arranged with uniform polarity in the synthetic filament.

At present, we reserve further speculations about the role of the myosin head CVD in producing myosin head power stroke, but would like to emphasize that a number of findings obtained using in vitro motility assay systems are interesting, but great care should be taken before relating the results obtained to the mechanism of muscle contraction consisting of hexagonal myofilament-lattice structure.

The discussions stated above, however, rest on the assumption that anti-RLR antibody molecule (IgG) does not bind to nitrocellrose memberane, on which in vitro actin-myosin sliding occurs. If, however, anti-RLR antibody molecule can bind to nitrocellurose membrane unlike the other two antibodies, it might act to inhibit actin-myosin sliding by crosslinking myosin heads to the substratum. This possibility should be kept in mind.

### Evidence for Essential Role of Junction between Myosin Head LD and Myosin Subfragment 2 in Producing Muscle Contraction

**Figure 10:**
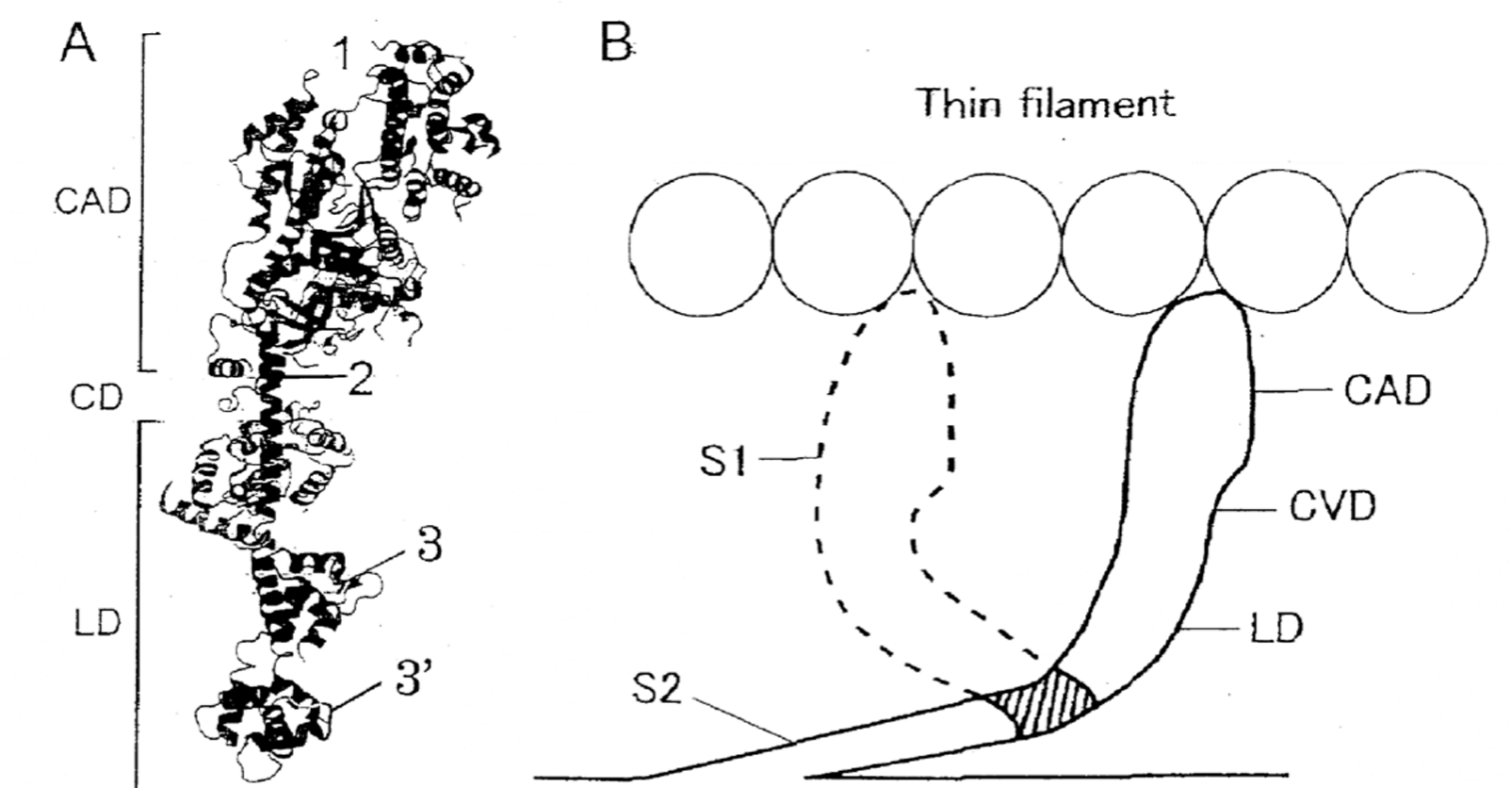
Schematic diagrams showing change in myosin head configuration before and after power stroke. (A) Ribbon diagram of the myosin head showing approximate regions of attachment of anti-CAD, anti-CVD and anti-LD antibodies, indicated by numbers 1, 2 and 3 and 3′, respectively. (B) Myosin head configurations before and after power stroke, indicated by solid and broken lines, respectively. Note rotation of the LD around the LD-S2 junction (shaded area) as well as rotation of the CAD around the CVD.

Figure 10A is a ribbon diagram of the myosin head in which approximate points of attachment of the three different antibodies used in the present experiments are indicated, while Figure 10B shows schematic representation of changes in myosin head configuration before (solid line) and after (broken line) power stroke. As can be seen in Fig. 10B, rotation of the LD around the LD-S2 junction takes place as well as rotation of the CAD around the CVD. Since anti-LD antibody attaches to the peptides of regulatory light chains near the LD-S2 junction, its inhibitory effect on Ca^2+^-activated force development (Figs.7–9) indicates involvement of this region in the rotation of the LD around the LD-S2 junction producing myosin head power stroke. The above idea may also be consistent with our unpublished observation that the velocity of actin filament sliding along synthetic “mini” myosin filaments with uniform polarity [34], is reduced by ∼50% with 0.2 mg/ml ant-LD antibody, and is reduced to zero with anti-LD antibody above 1 mg/ml; the complete elimination of actin filament sliding along “mini” myosin filament may result from that the antibody binds with all the myosin heads available for filament sliding. On the other hand, the ineffectiveness of anti-LD antibody on MgATPase activity of muscle fibers (Fig. 9) may be due to that the CAD, which contains ATP-binding site, is geographically distant from the LD-S2 junction.

The importance of the LD-S2 junction in producing muscle contraction has also been indicated by the report that a polyclonal antibody to the S2 region inhibits muscle fiber contraction without changing MgATPase activity [19]. On the other hand, the ineffectiveness of anti-LD antibody on in vitro actin-myosin sliding (Fig. 6) can also be accounted for by the manner of fixation of myosin heads on a glass surface, As myosin heads are truncated at the LD-S2 junction so that rotation of the LD around the LD-S2 junction can no longer take place. A small tendency of the actin-myosin sliding velocity might result from that, in a small proportion of fixed myosin heads, truncation is incomplete so that the LD rotation around the LD-S2 junction takes place.

Despite the reduction of the maximum Ca^2+^-activated isometric force by the antibody in a concentration-dependent manner (Fig. 7), the force-velocity curves were scaled according to the maximum force developed, while the V_max_ remained unchanged (Fig. 8A,B). These results indicate that myosin heads, in which rotation of the LD around the LD-S2 junction is impaired by the antibody, provides no appreciable internal resistance against muscle fiber shortening. This also implies that reduction in the number of myosin heads involved in producing contraction may constitute another reason for the reduction of isometric force by anti-LD antibody.

In addition to the structures essential for muscle contraction, including actin and nucleotide binding sites in the CAD and switches I and II linking these sites to the CVD [6], the present results indicate that the CAD-CVD junction and the LD-S2 junction also contribute in producing force and motion in muscle. Much more experimental work is necessary to clarify mechanisms of muscle contraction at the molecular level.
